# Proactive Personality as a Predictor of Career Adaptability and Career Growth Potential: A View From Conservation of Resources Theory

**DOI:** 10.3389/fpsyg.2021.699461

**Published:** 2021-09-09

**Authors:** Su Wang, Mei Mei, Yang Xie, Yiting Zhao, Fu Yang

**Affiliations:** ^1^School of Business Administration, Southwestern University of Finance and Economics, Chengdu, China; ^2^School of International Business, Southwestern University of Finance and Economics, Chengdu, China; ^3^School of Insurance, Southwestern University of Finance and Economics, Chengdu, China; ^4^School of Business Administration, Guizhou University of Finance and Economics, Guiyang, China

**Keywords:** proactive personality, friend support, teacher individualized consideration, emotional exhaustion, career adaptability, career growth potential

## Abstract

In the present study, we offered a new account for the development of career adaptability and the realization of career growth potential based on conservation of resources (COR) theory. Using data collected from 903 university students in China, we examined how and when proactive personality influences students’ career adaptability and career growth potential by introducing emotional exhaustion as a mediator as well as friend support and teacher individualized consideration as boundary conditions. Specifically, the results confirmed a positive effect of proactive personality on career adaptability, with this relationship mediated by emotional exhaustion. In addition, results suggested a positive effect of proactive personality on career growth potential, with this relationship mediated by emotional exhaustion and career adaptability. Moreover, results showed that in-school social support (i.e., friend support and teacher individualized consideration) served as moderators in the relationship between proactive personality and emotional exhaustion, such that the negative effect of proactive personality on emotional exhaustion was strengthened when students received high levels of social support. Theoretical implications of career adaptability research and COR theory and practical implications for promoting adaptability resources and career growth in university are provided.

## Introduction

The increasing boundarylessness and unpredictability in current career patterns require individuals to enhance their career adaptabilities to be competitive and adapt to the changing world (Uy et al., 2015). Proactive personality, which refers to an individual’s relatively stable tendency to take initiative to influence one’s surroundings (Bateman and Crant, 1993), has been considered a pivotal determinant for acquiring career adaptability resources and attaining good adaptation outcomes (e.g., Pan et al., 2018). Despite the clear importance of this construct in career development, research on the underlying mechanisms through which proactivity affects career adaptability and career-related outcomes is still rare. For this reason, the current study aims to propose and examine a comprehensive model of proactive personality and its influence on students’ career adaptability and subsequent career growth.

Along with proactivity, career adaptability, one of the central components of Career Construction Theory (Savickas, 2002), has been argued to be a critical variable in career development. Career adaptability is defined as the “readiness to cope with the predictable tasks of preparing for and participating in the work role and with the unpredictable adjustments prompted by changes in work and working conditions” (Savickas, 1997, p. 254). This construct has a wide range of positive occupational implications (for a review, see Johnston, 2016). It denotes individuals’ competencies and knowledge for coping with potentially stressful situations and challenges in their occupational roles (Savickas and Porfeli, 2012). Given the unparalleled changes in the global context and job market in recent years, it is important that university students acquire and develop their career adaptability resources to adapt to career demands (Hou et al., 2012; Shin and Lee, 2016).

To date, numerous studies have explored the predictors of career adaptability and the effectiveness of career adaptability in predicting positive career-related outcomes. Of these studies, majority have adopted a perspective from career construction theory (Savickas, 2005), arguing that the adaptive readiness, such as the Big Five (Zacher, 2014; Neureiter and Traut-Mattausch, 2017), core self-evaluation (Xu and Yu, 2019), and goal orientation (Tolentino et al., 2014), will affect the level of career adaptability and career adaptation. Although these studies have significantly extended our understanding of the predictors of career adaptability and the process of career construction, they ignored that other types of resources besides personal characteristics will also affect individuals’ resources loss or resource gain process. Conservation of resources (COR) theory (Hobfoll, 2001) suggests that individuals who have greater resources are likely to acquire more resources. To extend current knowledge about career adaptability, we draw upon the COR theory and consider career adaptability as resources that students can develop through resource conservation.

In order to explain the association between proactive personality and career adaptability based on a COR perspective, we attempt to explore the mediating effect of emotional exhaustion, the key component of burnout, referring to “a lack of energy and a feeling that one’s emotional resources are used up” (Cordes and Doughtery, 1993, p. 623). Past research has indicated that individuals who are exhausted often report unsatisfactory adaptation results such as cognitive impairments (Feuerhahn et al., 2013), decreased satisfaction at work (Bacharach et al., 1991), and intention to quit school (Koeske and Koeske, 1991). According to COR theory (Hobfoll, 1989, 2001), people tend to control and retain resources that they value. Those who have greater personal resources are more likely to experience resource gain, in turn, leading to a resource gain spiral. Consistent with this corollary, we propose that students with high levels of personal resources (i.e., proactive personality) may be less likely to deplete their emotional resources, thereby leading to higher levels of career adaptability resources.

Furthermore, as suggested by COR theory (Hobfoll, 2001), supportive context can be a kind of compensation that contributes to a maintenance of resource reservoirs and resource gains. In the present study, we focus on the moderating roles of two types of social support (i.e., friend support and individualized consideration from teachers) and theorize that being supported by friends and teachers can magnify the positive effect of proactive personality. Research has investigated the buffering role of supportive context in diminishing stress appraisal and thereby promoting health and wellbeing (Oliva et al., 2009; Thurman et al., 2018). By providing individuals with emotional and tangible support (Hirschi, 2009), supportive social relationships are significant resources in protecting individuals from resource loss and facilitating future resource gain (Hobfoll, 2001). As such, friend support and teacher individualized consideration are expected to strengthen the negative relationship between proactive personality and emotional exhaustion.

**FIGURE 1:**
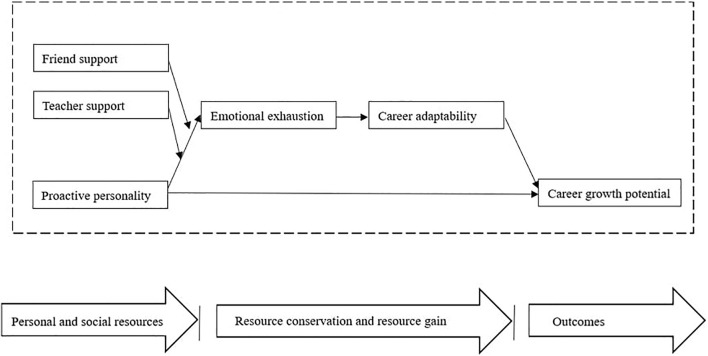
The theoretical model.

In sum, we adopt a new perspective (i.e., COR theory) to explain the association between university students’ proactivity and their career outcomes. Our research has four primary contributions to the extant literature. First, drawing on the perspective that proactive personality is a personal resource that may lead to further resource gain, we argue that proactive personality can increase individuals’ career adaptability resources, which in turn, should enable students to achieve higher levels of career growth potential. In this regard, we extend the extent literature on the antecedents of career growth potential and the process through which proactive personality predicts better adaptation outcomes. Second, although past research has demonstrated the positive relationship between proactive personality and career adaptability (e.g., Guan et al., 2017), still very few studies have explored the underlying mechanisms of proactive personality on the development of career adaptability (notable exceptions being Hou et al., 2014; Cai et al., 2015; Uy et al., 2015; Jiang, 2017). In the current research, we identify the mediating role of emotional exhaustion and argue that students in higher levels of proactive personality are less likely to become depleted. Thus, proactive students can increase their career adaptability resources through conservation of energy resources. Doing so is important because it provides a new insight for understanding the mechanism underlying the relationship between proactive personality and career adaptability. Third, we examine the moderating effect of in-school social support on emotional exhaustion. In this respect, our research offers an indication that individuals’ condition resources (e.g., friend support and teacher individualized consideration) would amplify the negative effect of personality resource (i.e., proactive personality) on subsequent resource loss (emotional exhaustion). Fourth, by demonstrating the serial mediating effects of emotional exhaustion and career adaptability on the relationship between proactive personality and career growth potential, we provide a more comprehensive understanding of how initial resources promote subsequent resource spirals. Figure 1 depicts the theoretical model.

## Theoretical Background and Hypothesis Development

### The Conservation of Resources Theory

According to Hobfoll (1988), resources refer to those objects, conditions, personal characteristics and energy and the methods to acquire them. These resources are specifically divided into different categories. Especially relevant for this research are the following three categories: conditional resources (such as qualifications and experience), personal resources (such as self-efficacy and self-esteem), and energy resources (such as knowledge and social support). COR theory combines resources with “goal” and indicates that resources are any material or condition that can help an individual achieve a goal or satisfy a need (Halbesleben et al., 2014).

COR theory follows from a basic tenet that individuals strive to obtain, retain, and foster those things that they are value (Hobfoll, 2001). Following this central tenet, COR theory posits a number of principles and corollaries. COR theory suggests that the impact of resource loss on the individuals is more salient compared with the resource gain. Individuals must invest more resources to avoid the continuous loss of resources or recover from the loss (Hobfoll, 2011), including direct replacement of resources and indirect investment in resources. Those who have limited resources are likely to experience resource loss, in turn, leading to a resource loss spiral (Hobfoll, 2001). In addition, Hobfoll et al. (2018) pointed out that resources do not exist individually. They are likely to be the consequence of nurturance and adaptation between individual and environment. This kind of environment can not only cultivate and nourish resources, but also limit and hinder the maintenance, development and creation of resources. As suggested by COR theory, supportive context can be a kind of compensation that contributes to a maintenance of resource reservoirs and resource gains (Hobfoll, 2001).

### Proactive Personality and Career Growth Potential

Proactive personality refers to the disposition to engage in proactive behaviors and to influence one’s own environments (Bateman and Crant, 1993). Compared with their counterparts with low proactivity, individuals with high proactivity are more likely to effect environmental change rather than being constrained by situational forces (Crant, 2000). Proactive individuals are normally change- and action-oriented (Jiang, 2017), enabling them to identify improvement opportunities, take actions, and persevere until they change and improve the status quo (Bateman and Crant, 1993; Crant, 2000). Career growth refers to “the process through which individuals develop new skills and capabilities and take on new responsibilities and enriched job roles to achieve future career goals and objectives” (Liu et al., 2010, p. 1437). Career growth potential reflects the probability of obtaining career growth opportunities and achieving one’s career goals. As mentioned above, proactive individuals actively identify opportunities and act on them (Bateman and Crant, 1993). They select and create environments that facilitate the realization of performance goals (Crant, 1995). Therefore, individuals with high levels of proactive personality are more inclined to facilitate career development and achieve good career outcomes (Jiang, 2017). Prior empirical evidence has suggested that proactive personality is a crucial individual difference variable related to favorable career outcomes such as career progression and career satisfaction (Seibert et al., 1999; Seibert et al., 2001), job performance (Crant, 1995; Thompson, 2005), and supervisor-rated career success (Yang and Chau, 2016). For these reasons, proactive personality may serve to increase the potential of career growth. Thus, we formulate the following hypothesis:

Hypothesis 1. Proactive personality is positively related to career growth potential.

### The Mediating Role of Emotional Exhaustion

Emotional exhaustion is characterized by a lack of energy resources and occurs when there is a depletion of one’s emotional resources (Maslach, 1993). When people feel their emotional resources are depleted, they are normally found to be associated with a variety of physical and psychological symptoms and negative interpersonal consequences (Cordes and Doughtery, 1993). COR theory suggests that individuals with less resources are less capable of resource gain (Hobfoll, 2001). As a result, emotionally exhausted individuals are less likely to develop their career resources due to the lack of energy. Moreover, scanning and utilizing opportunity for development or making progress toward one’s career goals require effort and energy. When a person feels that his or her emotional resources are used up, he or she are less likely to strive to develop their career skills, decreasing one’s career growth potential. Thus, we theorize that emotional exhaustion is related negatively to career growth potential.

However, proactive individuals who exert control over their work situations and careers are more likely to anticipate changes (Seibert et al., 1999) and save energy for future tasks. Given that resources enable one to improve his or her current and future situations, the more resources that are conserved, the less negative the outcomes one will experience (Watkins et al., 2014). Thus, individuals high in proactive personality are less vulnerable to emotional exhaustion, which in turn, should enhance the likelihood of achieving career growth. Based on previous arguments, we thus propose the following hypothesis:

Hypothesis 2. The relationship between proactive personality and career growth potential is mediated by emotional exhaustion.

### The Mediating Role of Career Adaptability

Career growth encapsulates need satisfaction such as obtaining opportunities for training and development, achieving professional ability development, and meeting career goals (Liu et al., 2010; Weng et al., 2010). In the career construction literature, career adaptability competencies have been empirically shown to be significant vocational resources that help individuals form adaptive strategies and achieve development and adaptation goals (Guan et al., 2013; Fiori et al., 2015; Celik and Storme, 2018). With career adaptabilities, individuals are likely to attain positive career outcomes such as career success (Yu et al., 2018), perceived internal and external marketability (Spurk et al., 2016), and subjective well-being (Maggiori et al., 2013). Consequently, career adaptability should serve to increase career growth potential. Furthermore, according to COR theory (Hobfoll, 2001), those who have greater resources are likely to experience resource gain spiral. Consistent with this argument, we propose that students who are high in proactivity are more likely to obtain and develop their adaptability resources, which in turn led to better career adaptation results. In sum, we propose the following hypothesis:

Hypothesis 3. The relationship between proactive personality and career growth potential is mediated by career adaptability.

### A Serial Mediation Effect

Individuals with high levels of proactive personality tend to actively deal with career-related problems, explore themselves and environments, and proactively take control of their surroundings to facilitate adjustment and career development (Kammeyermueller and Wanberg, 2003; Cai et al., 2015; Jiang, 2017). In terms of COR theory, those who with greater initial resources are less likely to lose resources and more likely to obtain resources (Hobfoll, 2001). Therefore, individuals high in proactivity are more capable of preventing emotional resource loss or developing their career-related resources. Indeed, scholars have argued that personal resources that facilitate initiative and control can reduce emotional exhaustion (Ito and Brotheridge, 2003), suggesting that proactive personality should have a direct negative effect on emotional exhaustion. Empirical studies have also demonstrated that having a high proactive personality will relate positively to one’s career adaptability resources (e.g., Hou et al., 2014; Tolentino et al., 2014; Uy et al., 2015; Guan et al., 2017; Jiang, 2017). In addition, taking hypotheses 2 and 3 together, emotional exhaustion and career adaptability may serve as a potential bridge between proactive personality and career growth potential.

Given the above theoretical and empirical evidence, we expected to theorize a serial mediation pattern where emotional exhaustion and career adaptability mediates the link between proactive personality and career growth potential. In particular, proactive personality can positively affect career adaptability via decreased emotional exhaustion. Career adaptability, in turn, may influence subsequent career growth potential by enhancing career preparedness and responsibilities to exert control in career development. Thus, we formulate the following hypothesis:

Hypothesis 4. The relationship between proactive personality and career growth potential is mediated by emotional exhaustion and career adaptability (serial mediation).

### The Moderating Role of Friend Support and Teacher Individualized Consideration

Social, or contextual, support is a multidimensional construct referring to instrumental and emotional environmental resources (Han and Rojewski, 2015), Of the social support that university students may experience, we pay close attention to the support from friends and teachers because past research has emphasized the importance of in-school social support in facilitating students’ emotional well-being (Griffin et al., 2019). As mentioned before, social support is an important element because it contributes to the maintenance of a strong resource repository (Hobfoll, 2001). Support from teachers and friends can be a powerful source of emotional and material resources that can be used in the process of career development (Hirschi, 2009; Han and Rojewski, 2015). Therefore, social support should serve as boundary conditions that strengthen the negative association between proactive personality and emotional exhaustion.

More specifically, the negative relationship between proactive personality and emotional exhaustion should be stronger when students’ perceived availability of friend and teacher support is high. When proactive individuals are engaged in career development and take action to make changes to the surroundings (Bateman and Crant, 1993), emotional and instrumental support from their important relationships should be critical resources that compensate for the consumption of energy and emotional resources, which in turn will lead to less emotional exhaustion. For example, research has reported that those who are supported by peers are more likely to effectively solve the developmental tasks in their careers (Kracke, 2002) and adapt better to school life (Berndt, 1999). In addition, teacher individualized consideration implies creating suitable conditions for students to play their own strengths (Li and Shi, 2005), and serves as a protective factor against negative outcomes among students with problems off campus (Thurman et al., 2018). Due to these reasons, proactive students can conserve their energy and make full use of their own dispositional advantages in a supportive context when striving for academic and career goals. By contrast, low friend and teacher support means that students should have less access to academic resources than those who are supported by friends, especially classmates, and teachers, making them more likely to appraise their career-related tasks as pressures and more vulnerable to academic failure (Domagała-Zyśk, 2006). Consequently, proactive individuals are more likely to be emotionally exhausted without friend and teacher support due to the consumption of resources to find more opportunities and take actions. In sum, we formulate the following hypotheses:

Hypothesis 5a. Friend support moderates the relationship between proactive personality and emotional exhaustion, such that the negative relationship is stronger for students who perceive high friend support than for those who perceive low friend support.

Hypothesis 5b. Teacher individualized consideration moderates the relationship between proactive personality and emotional exhaustion, such that the negative relationship is stronger for students who perceive high individualized consideration than for those who perceive low individualized consideration.

## Materials and Methods

### Participants and Procedures

Our participants were recruited from a large public university in Southwest China. This research has been approved by the Academic Ethics Committee of Southwestern University of Finance and Economics. Informed consent was obtained from all participants in this study. The first author collected research data with the assistance of class tutors and monitors using paper-based surveys. The participants were investigated by class units. Before the formal investigation, the first author approached the class tutors to explain the purposes of our project and obtain permission to collect the research data from the students. The class monitors also helped to publicize the investigation in the class. Then, the first author and his teaching assistants distributed the questionnaires to the students during class meetings. Before completing the questionnaire, the research team explained the study goals to the students. Written informed consent was obtained from all participants and the participation was ensured to be voluntary and all data were collected anonymously.

Students of different grades and degrees in this university are investigated. A total of 940 students responded to the survey. Some questionnaires were excluded from the final analysis because of either large number of missing responses or missing the rating of key variables. Finally, we received 903 valid responses from 35 classes (at least ten responses from each class), for a response rate of 96.1%. Of the 903 participants, 47.6% were male, and 57.4% were female. The majority (93.6%) were between 19 and 22 years old (SD = 0.28) and 96.7% were undergraduate students.

### Measures

With the exception of the individualized consideration scale, which was originally a Chinese version scale, all other scales used in the present study were originally developed in English. We translated these English version measures into Chinese following the conventional back translation procedure (Brislin et al., 1973). Unless otherwise indicated, all ratings were made on a five-point Likert-type scale ranging from 1 (*Strongly disagree*) to 5 (*Strongly agree*).

#### Proactive Personality

We measured proactive personality with the most widely used ten-item instrument (Seibert et al., 1999). This scale is a short version of Bateman and Crant’s (1993) Proactive Personality Scale. Sample items are, “I am always looking for better ways to do things,” “If I believe in an idea, no obstacle will prevent me from making it happen.” Cronbach’s alpha for this scale was 0.885.

#### Friend Support

To measure students’ perceived support from their friends, we used the four-item friend support subscale from the Scale of Perceived Social Support (Zimet et al., 1988). Sample items are, “My friends really try to help me,” “I can count on my friends when things go wrong.” Cronbach’s alpha for this scale was 0.809.

#### Individualized Consideration

We adapted the six-item individualized consideration subscale from the Chinese version of transformational leadership scale (Li and Shi, 2005). Sample items are, “My teacher will consider my actual situation when communicating with me,” “My teacher is willing to help me solve life and family problems.” Cronbach’s alpha for this scale was 0.929.

#### Emotional Exhaustion

We adapted three items from Boswell et al. (2004) to measure students’ emotional exhaustion. Sample items are, “I feel emotionally drained from my work and study in the university,” “I feel burned out from my work and study in the university.” Cronbach’s alpha for this scale was 0.856.

#### Career Adaptability

We measured career adaptability using the Career Adapt-Abilities Scale-China Form (Hou et al., 2012), which has been well validated among Chinese samples (e.g., Tian and Fan, 2014; Cai et al., 2015). This scale consists 24 items that are divided into four subscales: concern, control, curiosity, and confidence. Sample items are, “Thinking about what my future will be like” (concern), “Making decisions by myself” (control), “Exploring my surroundings” (curiosity), and “Performing tasks efficiently” (confidence). The Cronbach’s alpha for the overall scale was 0.944.

#### Career Growth Potential

The four-item scale developed by Wang et al. (2019) was used to measure career growth potential. Sample items are, “In the future, I can realize my career goals,” “In the future, I can gain career-related resources and opportunities.” Cronbach’s alpha for this scale was 0.884.

#### Control Variables

Following past research on career adaptability and career growth (e.g., Weng et al., 2010; Guan et al., 2017), we controlled for students’ age, gender and education in the current study. In addition, given that participation in student organizations will increase students’ career skills (e.g., networking), we also controlled for the experiences of students’ participating in student organizations using a dummy variable (1 = *have such experiences*, 0 = *no such experiences*).

## Results

### Confirmatory Factor Analysis and Descriptive Statistics

Descriptive statistics and correlations for study variables and controls are presented in Table 1. Prior to further analyses, we computed the variance inflation factor (VIF) for each independent variable. As the highest VIF is 2.424, less than the threshold 5 (Hair et al., 2011), which suggested no severe multicollinearity among the variables. We conducted confirmatory factor analysis (CFA) with MPLUS (Version 7.4; Muthén and Muthén, 2012) to examine the discriminant validity of the measurement models. The CFA results indicated that the hypothesized six-factor model showed a satisfactory model fit [*χ^2^*(419) = 2.97, CFI = 0.92, TLI = 0.91, RMSEA = 0.05, SRMR < 0.04], which is better than the alternative models (see Table 2). These results suggest empirical distinctions among the research variables.

**TABLE 1:** Means, standard deviations, and correlations among variables.

Variable	*M*	SD	1	2	3	4	5	6	7	8	9
1. Gender	1.574	0.495									
2. Age	2.010	0.280	−0.10**								
3. Education	2.963	0.205	0.01	−0.54**							
4. Experiences in student organizations	0.638	0.481	0.03	−0.05	−0.01						
5. Proactive personality	3.710	0.629	−0.05	−0.00	−0.02	0.02					
6. Peer support	4.178	0.741	0.08*	−0.01	0.02	0.08*	0.39**				
7. Teacher support	3.745	0.750	−0.03	0.05	−0.06	0.07	0.47**	0.42**			
8. Emotional exhaustion	2.733	0.958	−0.03	−0.07*	0.03	−0.04	−0.12**	−0.05	−0.11**		
9. Career adaptability	3.901	0.542	−0.02	0.01	−0.04	0.02	0.77**	0.50**	0.53**	−0.18**	
10. Career growth potential	3.924	0.658	−0.02	0.01	−0.01	0.06	0.54**	0.44**	0.45**	−0.15**	0.68**

*N = 903. Gender was coded “1” for male and “2” for female; Age was coded “1” for 18 years old or under, “2” for 19–22 years old, “3” for 23–25 years old, and “4” for over 25 years old; Education was coded “1” for doctor, “2” for master, and “3” for bachelor; and Experiences in student organizations were coded “1” for yes and “0” for no.*

*p<0.05, **p<0.01.

**TABLE 2:** Confirmatory factor analysis results for the measurement models.

Model	*χ^2^*	df	*χ^2^/*df	CFI	TLI	RMSEA	SRMR
Hypothesized six-factor model	1,244.432	419	2.970	0.92	0.91	0.05	0.04
Five-factor model A: PP, PS, TS, EE, CA + CGP	1,712.946	424	4.040	0.88	0.87	0.06	0.05
Five-factor model B: PP, PS + TS. EE, CA, CGP	2,091.908	424	4.934	0.85	0.83	0.07	0.07
Four-factor model A: PP, PS + TS, EE, CA + CGP	2,418.247	428	5.650	0.81	0.79	0.07	0.08
Four-factor model B: PP + PS + TS, EE, CA, CGP	3,485.897	428	8.145	0.71	0.68	0.09	0.09
Three-factor model: PP + PS + TP, EE, CA + CGP	3,859.068	431	8.954	0.67	0.65	0.09	0.09
Two-factor model: PS + TS, PP + EE + CA + CGP	3,504.874	433	8.094	0.71	0.69	0.09	0.09
One-factor model: PP + PS + TS + EE + CA + CGP	4,992.925	434	11.504	0.57	0.54	0.11	0.10

*N = 903. PP, proactive personality; PS, peer support; TS, teacher support; EE, emotional exhaustion; CA, career adaptability; and CGP, career growth potential.*

### Testing Mediation Effects

To examine our mediated model, we utilized the bootstrapping-based procedure with SPSS macro program developed by Hayes (2013). As shown in Table 3, the results of total effect of proactive personality on career growth potential supported Hypothesis 1 (total effect = 0.508, se = 0.042, and 95% CI = [0.425, 0.593]). The results of bootstrapping analyses (5,000 resamples) confirmed the mediation effect of emotional exhaustion on the relationship between proactive personality and career growth potential (indirect effect = 0.010, se = 0.006, and 95% CI = [0.001, 0.023]), supporting Hypothesis 2. The results in Table 3 also confirmed the mediation effect of career adaptability on the relationship between proactive personality and career growth potential (indirect effect = 0.510, se = 0.043, and 95% CI = [0.426, 0.596]), supporting Hypothesis 3. In addition, PROCESS analysis was conducted to test the serial mediation of the link between the proactive personality and career growth potential via emotional exhaustion and career adaptability. The results showed that a 95% bias-corrected bootstrap confident interval excluded zero (indirect effect = 0.006, se = 0.003, and 95% CI = [0.001, 0.014]). Therefore, Hypothesis 4 was supported.

**TABLE 3:** Results of PROCESS analysis.

Path	Indirect effect	se	95% CI (Lower, Upper)
PP→EE→CA	0.008	0.004	(0.002, 0.019)
PP→EE→CGP	0.010	0.006	(0.001, 0.023)
PP→CA→CGP	0.510	0.043	(0.426, 0.596)
PP→EE→CA→CGP	0.006	0.003	(0.001, 0.014)
Total effect	0.508	0.042	(0.425, 0.593)

*N = 903. Bootstrap sample size = 5,000. PP, proactive personality; PS, peer support; TS, teacher support; EE, emotional exhaustion; CA, career adaptability; and CGP, career growth potential.*

### Testing Moderation Effects

**FIGURE 2:**
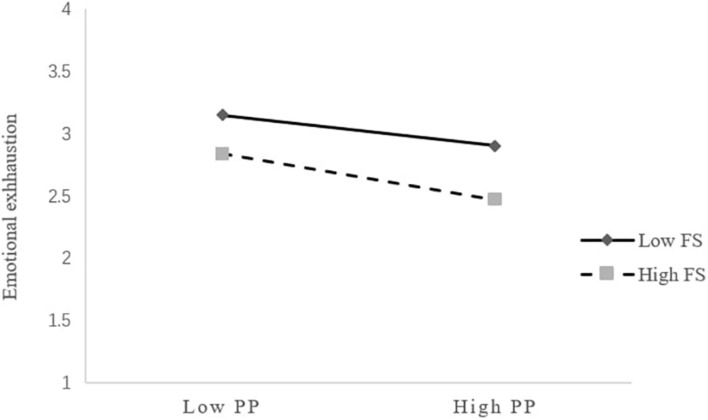
Interaction of proactive personality and friend support predicting emotional exhaustion. PP, proactive personality; FS, friend support.

We employed the commonly used three-step hierarchical regression analysis (e.g., Jiang, 2017) to test the moderating effects of friend support (Hypotheses 5a) and teacher individualized consideration (Hypotheses 5b) on the relationship between proactive personality and emotional exhaustion. Proactive personality, friend support and teacher individualized consideration were mean centered before entry into the regression equation (Aiken and West, 1991). As shown in Table 4, there was a significant interaction effect of proactive personality and friend support in predicting emotional exhaustion (*β* = −0.076, *p*<0.05). Figure 2 displays the interaction pattern for friend support. Whereas the relationship between proactive personality and emotional exhaustion was negative when friend support was low (simple slope = −0.127, 95% CI = [−0.245, −0.009], and *p*<0.05), the negative relationship became stronger when friend support was high (simple slope = −0.267, 95% CI = [−0.402, −0.132], and *p*<0.001). Thus, Hypothesis 5a was supported.

**TABLE 4:** Results of moderation effects of peer support and teacher support.

	Emotional exhaustion			
Variable	*M*1	*M*2	*M*3	*M*4
**Control variables**				
Gender	−0.03	−0.04	−0.04	−0.04
Age	−0.09*	−0.09*	−0.09*	−0.09*
Education	−0.02	−0.02	−0.02	−0.02
ESO	−0.04	−0.04	−0.04	−0.04
**Independent variable**				
Proactive personality		−0.12***	−0.13***	−0.10**
**Moderator**				
Peer support			−0.02	
Teacher support				−0.06
**Interaction**				
Proactive personality × Peer support			−0.08*	
Proactive personality × teacher support				−0.09**
**R^2^**	0.09	0.02	0.03	0.03
**ΔR^2^**	0.01	0.01***	0.01*	0.01**

*N = 903. ESO, Experiences in student organizations.*

*p<0.05, **p<0.01, and ***p<0.001.

**FIGURE 3:**
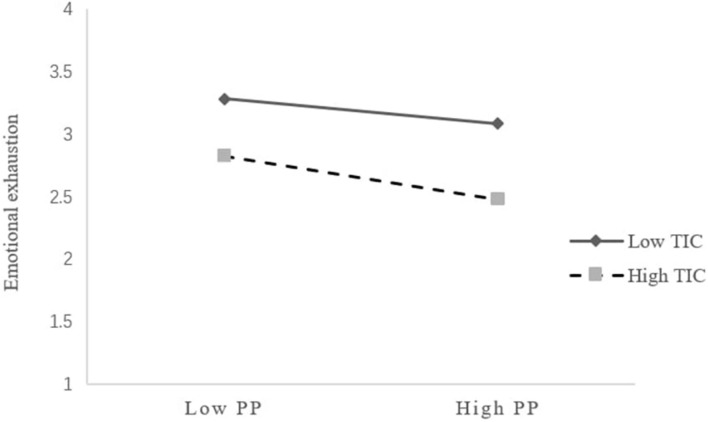
Interaction of proactive personality and teacher individualized consideration predicting emotional exhaustion. PP, proactive personality; TIC, teacher individualized consideration.

With regard to Hypothesis 5b, as shown by the results in Table 4, there was a significant interaction effect of proactive personality and teacher individualized consideration in predicting emotional exhaustion (*β* = −0.090, *p*<0.01). Figure 3 displays the interaction pattern for teacher individualized consideration. Consistent with Hypothesis 5b, the relationship between proactive personality and emotional exhaustion was significant and negative when teacher individualized consideration was high (simple slope = −0.251, 95% CI = [−0.391, −0.110], and *p*<0.001). However, the relationship became nonsignificant when teacher individualized consideration was low (simple slope = −0.067, 95% CI = [−0.190, 0.056], and *ns.*). Therefore, Hypothesis 5b was supported.

## Discussion

The present study examined how and why proactive personality may foster career growth potential. Our findings indicate that university students’ proactive personality is positively associated with career growth potential, with these relationships mediated by emotional exhaustion and career adaptability. In addition, we show that the negative relationship between proactivity and emotional exhaustion is more or less conditional on a high level of perceived in-school social support. These findings have important theoretical and practical implications.

### The Relation Between Proactive Personality, Career Adaptability and Career Growth

First and foremost, this study offers an alternative perspective (i.e., COR theory) to understand the association between proactive personality and career adaptability resources and subsequent career growth. COR theory (Hobfoll, 2001) posits that individuals who have greater initial resources are more likely to experience resource gain. In this regard, when individuals are high in proactivity, they are more likely to develop career adaptability resources. The increased career adaptability, which in turn, will lead to high potential of career growth. These findings are in accordance with previous research on the relationship between career adaptivity and adaptability and adaptation from a career construction perspective (e.g., Guan et al., 2017). In this respect, our study extends previous research on career construction literature by offering an additional theorical account to understand the association among career readiness, resources, and results (Savickas et al., 2009; Savickas and Porfeli, 2012).

Second, this study contributes to the career adaptability research by examining the mediating role of depletion of emotional resources from a resource conservation perspective. Extant research has predominantly examined the antecedents of career adaptability from a career construction perspective, suggesting that individual difference variables are related directly to career adaptability (e.g., Tolentino et al., 2014; Hirschi et al., 2015). To date, however, not much research has attempted to examine how individual personality affects the development of career adaptability. The findings in this study suggest that career adaptability is positively correlated with proactive personality, with this relationship mediated by conservation of emotional resources. This result responds to the expressed call for more attention to the underlying explanatory link between individual predictors and career adaptability (Cai et al., 2015). These findings also indicate the necessity to delve more deeply into how personal resources influence the development of career-related resources.

Third, in support of COR theory (Hobfoll, 2001), our test of the serial mediation effects of emotional exhaustion and career adaptability provide solid evidence that greater personal resources (i.e., proactive personality) facilitate emotional resource conservation and career adaptability resource gain, leading to subsequent higher levels of career growth potential. Existing research on career construction corroborates that career adaptability are the pivotal link between career adaptivity and adaptation results (e.g., Pan et al., 2018). However, empirical evidence is still rare when adaptivity, adaptability and adaptation variables are considered at the same time. This research extends previous knowledge about the life designing process (Savickas et al., 2009) from an alternative perspective. Also, our research offers valuable insights into how to make use of one’s own advantages to preserve and obtain more resources to promote career development.

### The Moderating Role of in-School Social Support

Another key contribution of the present study is to demonstrate that in-school social support (i.e., support from teachers and friends) is an important contingent factor for explaining the resource conservation process. Specifically, our results suggest that being supported by teachers and friends will foster the application of proactivity and amplified the negative association between proactive personality and emotional exhaustion. The investigation of moderating roles of friend support and teacher individualized consideration helps to understand the importance of social support as key resources for mitigating emotional resource depletion in the university context. These findings contribute to the research on the roles of specific dimensions of social support in different situations (e.g., Griffin et al., 2019).

### Implications, Limitations, and Suggestions for Future Research

The results in the present study have several important practical implications. Given that career adaptability plays an important role in predicting adaptive results (Guan et al., 2017), it would be helpful for educators and career counselors to understand which factors may affect the development of career adaptability resources when helping students realize successful careers. Our findings confirm the important roles of proactive personality in predicting career adaptability and subsequent career growth, and further suggest that emotional resources are the underlying mechanisms that link proactive personality to career adaptability and career growth. From a COR perspective, universities and practitioners could employ targeted strategies in order to effectively help students with different levels of proactive personality make progress toward their career development goals. For example, for students with high proactivity, universities can provide challenging and supportive environments to help proactive students make full use of their personal resources. For students with low proactivity, educators and counselors could implement relevant interventions, for instance, training for self-profiling and work exploration (Akkermans et al., 2015), to help them better know their resource reservoirs and available career-related opportunities for future career development.

In addition, the present study provides important implications regarding the role of social support in conserving emotional resources and promoting career adaptability. Our findings suggest that teacher individualized consideration is instrumental in amplifying the positive influence of proactive personality on students’ conservation of energy resources. Therefore, universities should emphasize the important role of teachers and selection and training programs are also needed in order to increase the level of teacher individualized consideration. Additionally, given that communication and coaching are important contents in individualized considerations (Li and Shi, 2005), it would be beneficial to create a good communication climate and feedback channels for supporting students. Furthermore, the present study found that friends play significant roles in decreasing students’ emotional exhaustion. Accordingly, it is worthwhile for universities to create opportunities for students to develop friendships. For example, universities can organize some informal communication activities to increase the interaction between students and their peers.

Several limitations in the present study need to be addressed. First, because we collected data only from a university in Southwest China, the generalizability of the results is limited. Future research should replicate the present study in other cultural settings and employee samples to increase the generalizability of the findings. In addition, the single source of data used in the present study may increase the risk of common method bias (Podsakoff et al., 2003), future research is encouraged to use multisource or multivalve data. Second, a cross-sectional design cannot make causal inferences. Although many studies adopting a career construction perspective indicate that proactive personality is a predictor of career adaptability (e.g., Hou et al., 2014; Cai et al., 2015; Jiang, 2017), other scholars argue that a reciprocal relationship may occur between proactive personality and work characteristics (Li et al., 2014). In this respect, it is possible that students’ initial levels of career adaptability will influence their proactivity. Thus, future research may consider employing a longitudinal approach to test the causality between proactive personality and career adaptability over time. Third, the present study explored the effect of three different resources (i.e., personal resources, social support, and emotional resources) on the development of adaptability resources and subsequent career growth, future research could investigate the effects of other important resources on career development. For example, object resources such as time for adequate sleep (Hobfoll, 2001) have been suggested to be important for the acquisition and maintenance of mental and physiological adaptation in varies domains (Åkerstedt, 2006; Weinberger et al., 2018). This would be an interesting avenue for future research to explore other types of resources that contribute to the career construction process, which should enhance our knowledge of the predictors of career adaptability and adaptation results.

## Conclusion

In sum, the current study demonstrated that university students’ proactive personality is positively associated with career adaptability and career growth potential, which extends the knowledge about the antecedents of career adaptability based on a COR perspective. Our findings highlight the importance of supportive context (i.e., teacher and friend support) for reducing energy depletion, and the theoretical insights gained through this study indicate the necessity to dig deeper to investigate the wide range of antecedents of career adaptability.

## Data Availability Statement

The raw data supporting the conclusions of this article will be made available by the authors, without undue reservation.

## Ethics Statement

The studies involving human participants were reviewed and approved by the Academic Ethics Committee of Southwestern University of Finance and Economics. The patients/participants provided their written informed consent to participate in this study.

## Author Contributions

SW and FY designed the study. SW drafted the manuscript and coordinated the data collection. MM analyzed the data and revised the manuscript. YX and YZ reviewed and revised the manuscript. All authors participated in the discussion of the manuscript revision.

## Conflict of Interest

The authors declare that the research was conducted in the absence of any commercial or financial relationships that could be construed as a potential conflict of interest.

## Publisher’s Note

All claims expressed in this article are solely those of the authors and do not necessarily represent those of their affiliated organizations, or those of the publisher, the editors and the reviewers. Any product that may be evaluated in this article, or claim that may be made by its manufacturer, is not guaranteed or endorsed by the publisher.
